# Oligomers and Polymers Based on Pentacene Building Blocks

**DOI:** 10.3390/ma3042772

**Published:** 2010-04-14

**Authors:** Dan Lehnherr, Rik R. Tykwinski

**Affiliations:** 1 Department of Chemistry, University of Alberta, Edmonton, Alberta, T6G 2G2, Canada; E-Mail: dan.lehnherr@ualberta.ca (D.L.); 2 Institut für Organische Chemie, Friedrich-Alexander-Universität Erlangen-Nürnberg, Henkestrasse 42, D-91054 Erlangen, Germany

**Keywords:** pentacene, oligomer, polymer, oligoacene, polycyclic aromatic hydrocarbon, semiconductor

## Abstract

Functionalized pentacene derivatives continue to provide unique materials for organic semiconductor applications. Although oligomers and polymers based on pentacene building blocks remain quite rare, recent synthetic achievements have provided a number of examples with varied structural motifs. This review highlights recent work in this area and, when possible, contrasts the properties of defined-length pentacene oligomers to those of mono- and polymeric systems.

## 1. Introduction

Over the past few years, significantly improved optoelectronic properties have been achieved by conjugating chromophores such as thiophenes [1,2] and anthracenes [3,4,5,6,7,8,9,10,11] to form oligomers and polymers. For example, conjugated oligomers and polymers of anthracene have promising charge-carrier mobilities and continue to be explored [3,4,5,6,7,8,9,10,11], even though anthracene is not regarded as a particularly good organic semiconductor for thin film applications. On the other hand, pentacene, a benzannulated relative of anthracene in the linear oligoacene family, has technologically relevant charge-carrier mobilities. Thus a logical hypothesis is that conjugated (and perhaps non-conjugated) oligomers and polymers of pentacene should provide further improved semiconductive materials.

Organic materials can provide numerous advantages over their inorganic counterparts, including ease of tunability of the HOMO and LUMO levels through chemical functionalization, compatibility with flexible substrates, as well as lower manufacturing costs. Today the field of organic semiconductors seems on the doorstep of revolutionizing applications in the areas of field effect transistors [12], light emitting diodes [13], sensors [14], thin film transistors [15], solar cells [16], and beyond. The use of polycyclic aromatic hydrocarbons (PAHs), such as pentacene, has figured prominently in these efforts. Unfortunately, pentacene faces problems of poor stability and low solubility in organic solvents, even though it has large charge-carrier mobilities [17,18,19]. The issue of stability is related to the fact that pentacene is easily oxidized in air, whereas the solubility issues derive from the strong intermolecular forces associated with π-stacking in a herringbone manner [17,18,19]. These issues cause problems for the processing of pentacene, including purification and deposition of pentacene films from solution for device fabrication. As a result, formation of pentacene-based devices typically requires the use of thermal vapor deposition. Historically, efforts to enhance the performance of pentacene-based devices have been through device fabrication techniques rather than synthetic derivatization of pentacene. However, synthetic advancements in the last 10 years have provided methodology for functionalizing pentacene toward the realization of solution-processable, semiconductive materials [17,18,19]. 

**Figure 1:**
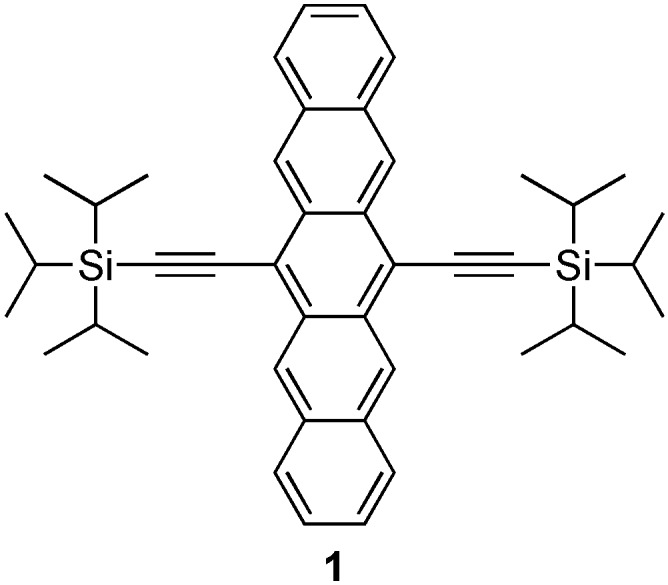
Structure of 6,13-bis(triisopropylsilylethynyl)pentacene (**1**).

One could not begin discussing functionalized pentacenes without acknowledging the discovery of 6,13-bis(triisopropylsilylethynyl)pentacene (**1**, Figure 1) by Anthony in 2001 [20]. Pentacene **1** is both benchtop stable and highly soluble in common organic solvents, which allows for easy processing, including purification and solution deposition of thin films for device fabrication. Additionally, the size of the trialkylsilyl groups allows for control of the solid-state packing [21] by interfering with the tendency of pentacene to stack in a herringbone manner. Thus, pentacene **1** adopts a highly beneficial face-to-face π-stacking in the form of a 2-dimensional bricklayer arrangement with short interplanar distances (3.47 Å) between the acenes, which facilitates electronic coupling in the solid-state. Despite almost a decade of intense research in the field of functionalized pentacene, pentacene **1** still stands among the best functionalized pentacene materials from a device fabrication stand point. The use of silylacetylenes has also been useful for the functionalization of larger acenes, e.g., hexacenes [22] and heptacenes [22,23], as well as smaller acenes like tetracene [24], anthracene [10,25], anthradithiophenes [26], and related chromophores [27,28]. Advances in the synthesis and study of functionalized pentacenes has been reviewed [17,18,19], and will not be discussed further. The focus of this review will be the synthesis and properties of oligomers and polymers based on pentacene building blocks.

## 2. Non-Conjugated Oligomers and Polymers

### 2.1. Non-conjugated Pentacene Oligomers

**Scheme 1:**
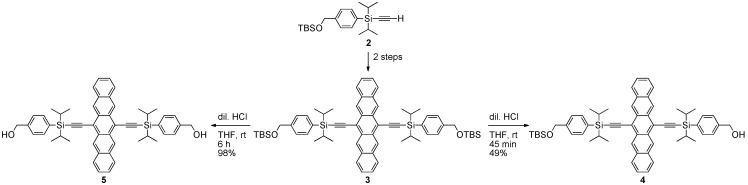
Pentacene building blocks **4** and **5** used for non-conjugated pentacene oligomers.

The first reported synthesis of pentacene-based oligomers dates back to 2007, when Tykwinski and Lehnherr reported a homologous series of non-conjugated pentacene dimers and trimers, as well as the corresponding polymers (see Section 2.2) [29,30]. Silylacetylene **2** was appended onto the pentacene chromophore in two steps from 6,13-pentacenequinone to afford **3** (Scheme 1). From pentacene **3**, building blocks **4** and **5** were obtained via removal of either one or both of the *tert*-butyldimethylsilyl (TBS) groups.

**Scheme 2:**
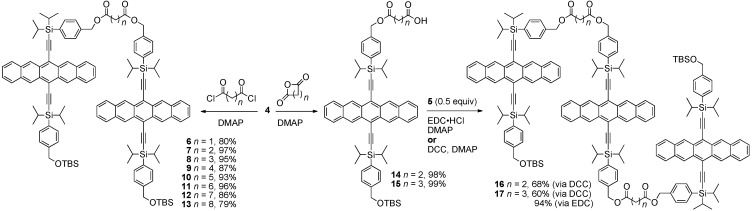
Synthesis of non-conjugated pentacene oligomers connected via ester linkages.

Pentacene dimers **6–13** were then obtained in good yield from **4** by simple esterification of a bis-acid chloride (Scheme 2). Pentacene trimers were also formed from alcohol **4** via initial reaction with cyclic anhydrides to form intermediates **14** and **15**. The pendent carboxylic acid functionality of **14** and **15** was then esterified via the reaction with **5** to provide trimers **16** and **17**, respectively. The esterification process could be accomplished with DCC, but higher yields and easier purification were possible with EDC·HCl.

Solution-state aggregation is observed for oligomers **6**, **7**, and **10** by ^1^H NMR spectroscopy at concentrations >0.05 M in CDCl_3_. Under dilute conditions (6.6 × 10^–7^ to 1.7 × 10^–5^ M), however, no aggregation is observed by UV–vis spectroscopy in CH_2_Cl_2_. Furthermore, no change in the qualitative shape or absorption maxima (±1 nm) is found for the UV–vis spectra of dimers **6–13** and trimers **16–17** compared to monomeric pentacenes **3–5**. Molar absorptivities (*ε*) increase monotonically as a function of the number of pentacene units: dimers **6–13** have roughly twice the *ε* values of monomer **5**, while trimers **16–17** have *ε* values three times that of **5** (comparison made at λ_max_ = 645 nm). Fluorescence studies using an excitation wavelength (λ_exc_) of 551 nm show that the emission maximum, located at 652 nm, is identical for all dimers and trimers. Fluorescence quantum yield (Φ_F_) studies show that dimers **6–13** all show quantum yields Φ_F_ = 0.08–0.11, whereas the values for trimers **16–17** (Φ_F_ = 0.06) are about half that of the dimers. Thermal analysis by differential scanning calorimetry (DSC) shows that all dimers and trimers begin to decompose somewhere in the range of 360–380 °C, and TGA analysis reveals no significant weight loss below 370 °C. Charge transport properties have unfortunately not been reported for these oligomeric materials.

### 2.2. Non-conjugated Pentacene Polymers

**Scheme 3:**
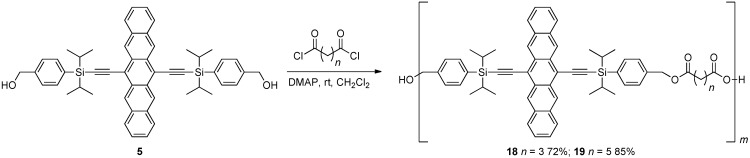
Synthesis of non-conjugated pentacene polymers **18–19** connected via ester linkages.

In addition to the oligomers described above, Tykwinski and Lehnherr reported non-conjugated pentacene-based polymers **18** and **19**, synthesized by reacting diol **5** with the acid chloride of either glutaric or adipic acid, respectively (Scheme 3) [29,30]. Although no weight-average molecular weight (*M*_w_) or number-average molecular weight (*M*_n_) were reported for these polymers, MALDI MS analysis showed macromolecules of over *m*/*z* 17,000 (e.g., *m* = 20) for **18** and *m*/*z* 15,000 (e.g., *m* = 17) for **19**. In addition to the generalized structure in Scheme 3, MALDI MS analysis also showed signals consistent with both termini of the polymer functionalized with either a carboxylic acid or an alcohol, as well as signals corresponding to macrocyclic structures. 

UV–vis absorption and emission characteristics for **18** and **19** are qualitatively equivalent (λ_max_ = 645 nm, λ_max,em_ = 652 nm) to those of the corresponding mono-, di- and trimers (*vide supra*). These polymers are soluble in solvents such as CH_2_Cl_2_, CHCl_3_, and THF and can be handled in the presence of air and water without any noticeable decomposition. Thin films cast from solution exhibit only a slight red-shift in λ_max_ (<10 nm) in comparison to their solution-state UV–vis absorptions. DSC and TGA analysis of polymers **18–19** show thermal stabilities comparable to dimers **6–13** and trimers **16–17**. 

### 2.3. Non-Conjugated Pentacene Dendrimers

Dendrimers are branched, 3-dimensional, defined-length oligomers and have been synthetically targeted for a range of applications, such as biomedical [31], catalysis [32], and light-harvesting [33,34]. Recently, the use of dendrimers as semiconductive materials for light-emitting diodes and photovoltaic cells has come to the forefront [35]. The dendritic structure offers the potential of enhanced performance through the ability to control the local environment of the chromophore within the macromolecular shell, as well as providing 3-dimensional ordering of the chromophores [36]. Since 3-dimensional arrangement of chromophores in the solid-state strongly influences the electronic properties of a material, and thus device performance, dendritic pentacene-based molecules might afford new and enhanced properties as a result of this controlled geometrical organization [17,18,19]. 

**Scheme 4:**
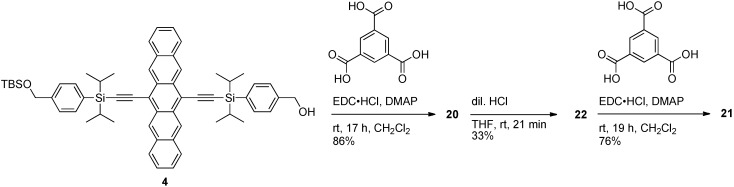
Synthesis of pentacene-based dendrimers.

**Figure 2:**
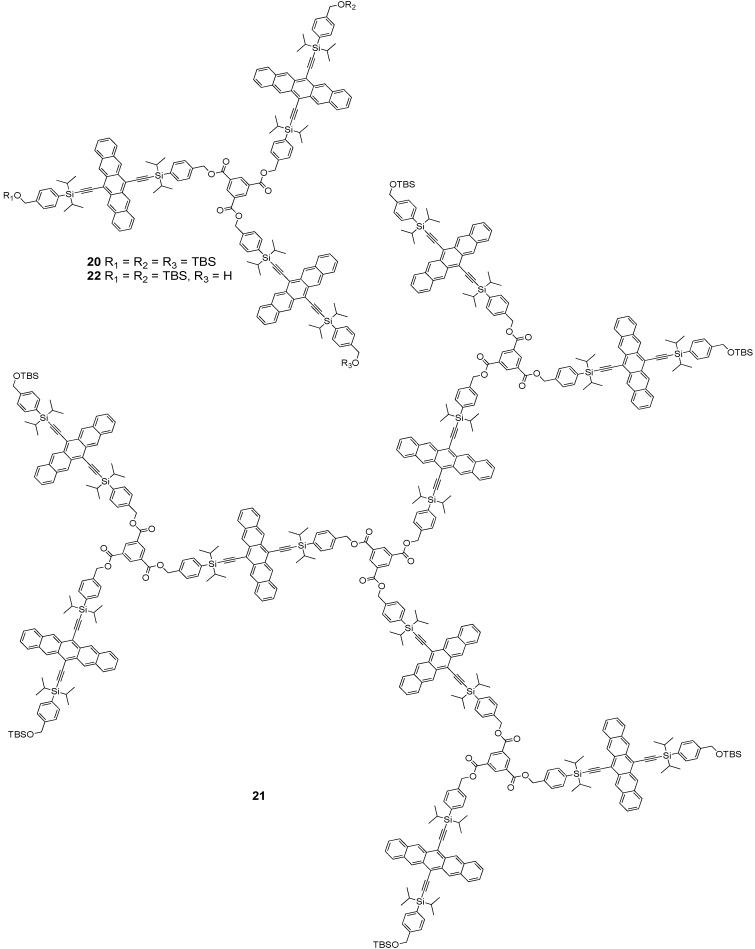
Structures of dendrimers **20** and **21**, and dendron **22**.

In 2009, Tykwinski and Lehnherr reported the synthesis and properties of pentacene-based dendrimers **20** and **21** (Figure 2) [37]. The esterification reaction of 1,3,5-benzenetricarboxylic acid with building block **4** in the presence of EDC provided dendrimer **20** (C_183_H_204_O_9_Si_9_, 2800 g·mol^–1^) in 86% yield (Scheme 4). A mild and carefully controlled desilylation of **20** with dilute HCl provided dendron **22**, which was then allowed to react with 1,3,5-benzenetricarboxylic acid in the presence of EDC to afford dendrimer **21** (C_540_H_570_O_30_Si_24_, 8214 g·mol^–1^) in 76% yield.

**Figure 3:**
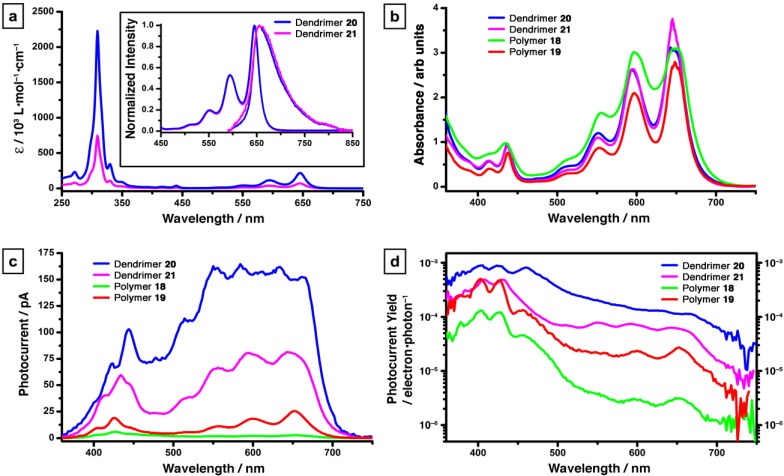
(a) Solution-state absorption and emission spectra for dendrimers **20–21** (in CH_2_Cl_2_). Plots of (b) UV–vis absorption spectra, (c) photoconductive response, and (d) photoconductive yield of thin film devices of dendrimers **20**–**21** and polymers **18–19**. Figure adapted with permission from reference [37]. Copyright 2009 American Chemical Society.

 Both **20** and **21** show significant solubility in solvents such as CH_2_Cl_2_, CHCl_3_, and THF. Similar to their linearly arranged cousins (oligomers **6–13** and **16–17**), no qualitative change in the UV–vis absorption profile for the dendrimers is found in comparison to monomer **5** (Figure 3a). The molar absorptivity of dendrimer **21** (*ε* = 2,230,000) for the most intense absorption (309 nm) is approximately three times larger than that of **20** (*ε* = 747,000), as expected based on the number of pentacene chromophores in each molecule. Solution-state fluorescence reveals a small Stokes shift of only 213 cm^–1^ for both dendrimers, suggesting minimal molecular rearrangement upon photoexcitation. Although the fluorescence spectra of dendrimers **20** and **21** appear similar to that of monomeric building block **5** (see Scheme 1 for structure), the fluorescence quantum yields in CH_2_Cl_2_ decrease as a result of dendrimer formation, Φ_F_ = 0.14 (monomer **5**) to Φ_F_ = 0.04 (**20**) to Φ_F_ = 0.03 (**21**). This trend is analogous to that observed in pentacene-based oligomers **6–13** and **16–17** (*vide supra*).

Thin film photodetectors have been formed using polymers **18–19** and dendrimers **20–21** by solution spin-casting. A specific element of this study examined photocurrent yield [38], which is defined as the ratio between the photogenerated electron flow rate and the absorbed photon rate given as:

Photocurrent yield = (*I*/*e*)/(*P*_abs_/*h*ν)

where *I* is the photocurrent, *e* is the electron charge, *P*_abs_ is the absorbed light power in the active region between the electrodes, *h* is Planck's constant and ν is the frequency of the light.

Dendrimers **20** and **21** show photoconduction onset at ca. 700 nm (Figure 3c) corresponding to the absorption edge for these materials (Figure 3b). This might indicate the presences of a band-to-band photogeneration mechanism [39], as opposed to an excitonic (Onsager) model [40,41]. Photocurrent yields for dendrimer **20** and **21** have been measured at 9 × 10^–4^ and 4 × 10^–4^, respectively (Figure 3d). These efficiencies are up to an order of magnitude greater than those of linearly-connected polymers **18** and **19**. It is worth noting that a flatter response in the photocurrent yield as a function of wavelength is also observed for the dendritic derivatives in comparison to the linear polymers. The photocurrent yield for dendrimers **20** and **21** varies approximately one order of magnitude over the range of 360–650 nm, while that of polymers **18** and **19** decreases by almost two orders of magnitude over the same range (Figure 3d). These results show that these dendritic materials might have enhanced efficiency compared to linearly-connected pentacene-materials, although the photocurrent yields are still very low. This is almost certainly due to a lack of effective π-stacking interaction between neighboring pentacene chromophores, a premise supported by the lack of any substantial red-shift in absorptions in the solid-state UV–vis spectra (Figure 3b) for **20**, **21** as compared to the solution-state data (Figure 3a).

## 3. Pentacene-Based Polycyclic Aromatic Hydrocarbon Conjugates as Pseudo-Oligomers

### 3.1. Arylated Pentacenes

The past decade has provided a large number of substituted pentacene derivatives, partly in attempt to gain understanding towards tuning the electronic properties of pentacene. The appendage of one or more aromatic chromophores to the pentacene core has been investigated, resulting in materials that could be described as pentacene-based PAH conjugates. While this review intends to cover pentacene oligomers and pseudo-oligomers comprehensively, it will not attempt to cover monomeric pentacene derivatives exhaustively. Substitution of pentacene with mononuclear aromatic rings such as substituted-benzene or thiophene systems either directly onto the pentacene or connected through an ethynyl linker will not be discussed in great detail as the topic is simply too large, instead we will briefly highlight some key points.

**Figure 4:**
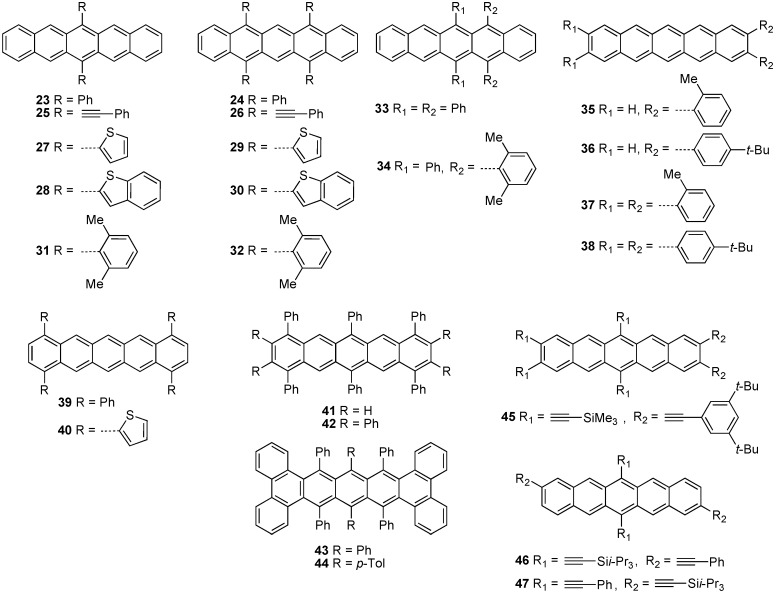
Arylated pentacene derivatives **23–47**.

In 1942, Allen and Bell synthesized 6,13-diphenylpentacene (**23**) and 5,7,12,14-tetraphenyl-pentacene (**24**) via addition of phenyl Grignard into 6,13-pentacenequinone and 5,7,12,14-pentacene-diquinone, respectively, followed by reductive aromatization using KI and acetic acid (Figure 4) [42]. This synthetic protocol, after some minor modifications, such as using SnCl_2_·2H_2_O in the presence of acid for the aromatization step, has provided the synthetic route to most 6,13-disubstituted pentacenes reported to date. In 1969, Maulding and Roberts reported the synthesis of 6,13-bis(phenylethynyl)pentacene (**25**) and 5,7,12,14-tetrakis(phenylethynyl)pentacene (**26**) from the corresponding acene mono- or diquinone [43].

In 2005, Dehaen and coworkers described the synthesis of 6,13-di(2'-thienyl)pentacene (**27**) and its benzothiophene analog (**28**) [44], but neither electronic properties nor device characterization were reported. However, Nuckolls and coworkers have studied the application of **27** for solar cells, in which **27** provided peak power efficiency of 1.4% when used in conjunction with PEDOT:PSS and C_60_ [45]. In 2006, Dehaen and coworkers also reported tetra-substituted pentacene derivatives **29** and **30** [46]. Sterically bulky aromatic rings such as 2',6'-dimethylphenyl group(s) have been attached to the pentacene core, such as in **31** [47] and **32** [48]. Kafafi has also reported 5,6,13,14-tetraarylated pentacenes **33** and **34** and their organic light emitting diode (OLED) device performance in 2006 [49]. In 2008, Neckers and coworkers reported the photochemical synthesis of di- and tetra-arylated pentacenes (**35–38**) at the pro-*cata* positions (2,3,9,10-positions of pentacene) [50]. Yamada and coworkers have developed a photochemical synthesis to 1,4,8,11-tetraarylpentacenes **39** and **40** [51,52]. Other substitution patterns have been achieved such as 1,4,6,8,11,13-hexaphenylpentacene (**41**) and 1,2,3,4,6,8,9,10,11,13-decaphenylpentacene (**42**) reported by Nuckolls [53] via generation of a naphthyne followed by *in situ* trapping with a diazopyrone. Recently, Pascal has reported several highly arylated pentacene derivatives (**43–44**) which have exceptionally twisted frameworks, as high as 144° for **43** [54,55].

In 2006, Neckers reported hexaethynylated pentacenes such as **45** which had a reduced electrochemical band gap (*E*_g_^electro^) of 1.69 eV in CH_2_Cl_2_ [56] and significantly red-shifted UV–vis absorption λ_max_ (681 nm in CH_2_Cl_2_) compared to 6,13-bis(triisopropylsilylethynyl)pentacene **1** (*E*_g_^electro^ = 1.81 eV, λ_max_ = 643 nm in CH_2_Cl_2_) [57]. In 2007, Fallis and coworkers reported tetraethynylated pentacenes such as **46** and **47** (λ_max_ ca. 650 nm in hexanes) [58]. Several groups have also tried to vary the electronic nature of the substituents on pentacene to explore various properties [59,60,61,62,63], including the influence on stability [48,64,65]. Even so, the impact of various substitution patterns on stability as well as the mechanism of photodegradation are still not well understood [48,66].

**Figure 5:**
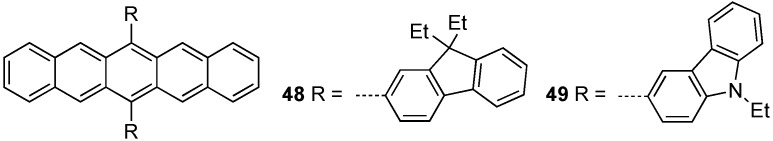
Structures of 6,13-bis(9,9-diethyl-9*H*-fluoren-2-yl)pentacene (**48**) and 6,13-bis(9-ethyl-9*H*-carbazol-3-yl)pentacene (**49**).

Larger aromatic systems have been appended onto the pentacene framework. In 2006, Park and coworkers reported pentacenes **48–49** functionalized with either fluorenes or carbazoles at the 6,13-positions of pentacene (Figure 5) [67]. These were synthesized via the typical addition of lithiated nucleophiles into 6,13-pentacenequinone followed by Sn^II^-mediated reductive aromatization of the resulting diol. The functionalization of the pentacene chromophore with these aromatic systems did not yield any change or red shift in the typical absorptions of pentacene [68], likely due to the inability for the side groups to be co-planar with the pentacene moiety as a result of steric interactions enforcing a twisted geometry. However, additional absorptions were observed in the UV–vis spectra corresponding to the carbazole and fluorene chromophores. The overall spectra of **48–49** overlap well with the photoluminescence spectra of tris(quinolin-8-olato)aluminum (III) (Alq_3_) making it a suitable host material for electroluminescent (EL) device fabrication with pentacenes **48–49**.

EL devices were fabricated using vacuum-deposition of pentacenes **48** onto ITO. EL devices had the following configuration: ITO/*m*-MTDATA/NPB/Alq_3_:**48** or **49** (5%)/LiF/Al (*m*-MTDATA = 4,4',4''-tris[*N*-(3-methylphenyl)-*N*-phenylamino)triphenylamine; NPB = *N,N'*-bis(1-naphthyl)-*N*,*N'*-diphenyl-1,1'-biphenyl-4,4'-diamine). Devices using pentacene **48** as a dopant showed red EL spectrum at 636 nm with 0.03 cd/A efficiency, while those using pentacene **49** showed dramatically better performances of 0.21 cd/A. These values are still both lower than those of devices with 6,13-diphenylpentacene (**23**) using 0.55 mol% pentacene:Alq_3_ which have 1.2 cd/A performance as reported by Kafafi and coworkers [69].

**Figure 6:**
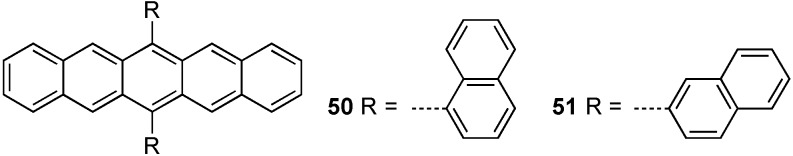
Structures of 6,13-bis(naphthyl)pentacenes **50–51**.

A variety of other PAH systems have been appended to pentacene, such as naphthyls (**50–51**) reported by Park (Figure 6) [70,71,72]. The 1-naphthyl isomer **50** had a higher melting point (272 °C) compared to the 2-naphthyl isomer **51** (160 °C) [71]. Both materials had photoluminescence maximum wavelengths around 620 and 660 nm [70]. OLED devices were fabricated using these pentacenes with the following device geometry: ITO/2-TNATA/NPB/Alq_3_ doped with either **50** or **51**/Alq_3_/LiF/Al in which electroluminescent wavelength maximum values were at 621 and 628 nm, respectively (2-TNATA = 4,4',4''-tris[*N*-2-naphthyl-*N*-phenylamino)triphenylamine) [72]. The EL efficiency of **51** (0.67 cd/A) was significantly higher than that of **50** (0.027 cd/A).

**Scheme 5:**
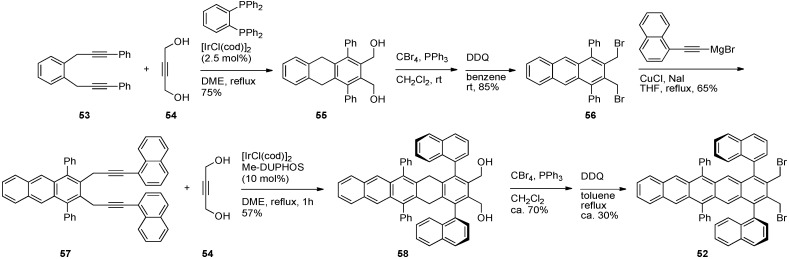
Synthesis of axially chiral pentacene **52**.

Naphthyl moieties have been appended to the pentacene skeleton at the 5,14-positions in the form of pentacene **52** (Scheme 5) [73]. *ortho*-Substituted benzene **53** was subjected to a iridium-catalyzed [2+2+2] cycloaddition with buta-2-yne-1,4-diol (**54**) to afford tricyclic **55**. Conversion of the alcohols of **55** to bromides followed treatment with DDQ to aromatize the system afforded anthracene dibromide **56**. Alkynylation of **56** with 1-ethynylnaphthyl Grignard provided intermediate **57**. A second iteration of the homologation procedure, in this case with Me-DUPHOS, was applied to anthracene **57** to afford pentacene **52** via intermediate **58**. Pentacene **52** has axial chirality and was synthesized in >99% ee. This synthetic approach is similar to Takahashi's homologation approach to pentacenes which uses a zirconium-mediated [2+2+2] cycloaddition of diynes [74,75].

**Figure 7:**
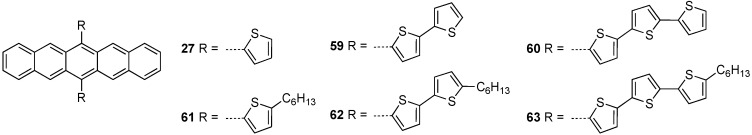
Structure of 6,13-bis(oligothienyl)pentacenes.

In 2009, Zhang and coworkers have reported the synthesis of pentacenes substituted with oligothiophenes at the 6,13-positions (Figure 7), as well as their anthradithiophenes analogs [76]. The energy level of the HOMO of these materials increases as a function of the oligothiophene chain length, for example in the alkylated series: –5.22 eV (**61**) to –5.14 eV for (**63**) (all *vs.* vacuum), which is consistent with increasing the effective conjugation length (Table 1). As for the LUMO energy, it decreases in energy as the number of thiophene units increases (from –3.14 eV (**61**) to –3.35 eV (**63**). The overall effect is a reduced electrochemical band gap, from 2.08 eV (**61**) to 1.79 eV (**63**), as the conjugation length increases with the length of the oligothiophene side chain. Analysis of the thermal stability (determined via TGA) as well as photostability of these hybrids affords no real trends as a function of the number of thiophenes in the side chain, although the alkylated thiophenes (**61–63**) are consistently more stable than their unalkylated counterparts. Increasing the number of thiophene units in the side chain results in a progressive decrease in solubility, although the alkylated derivatives have improved solubility compared to the alkyl-free derivatives. Solid-state packing of **62**, determined by X-ray crystallography, reveals a 1-dimensional slipped-stack arrangement with interplanar distance of 3.41 Å between the pentacenes.

**Scheme 6:**
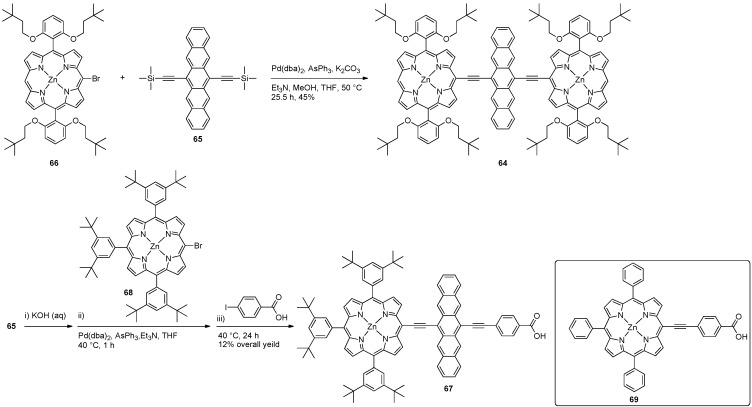
Synthesis of porphyrin functionalized pentacenes **64** and **67**. Inset: Model compound **69**.

In 2005, Therien and coworkers reported the synthesis of conjugated bis[(porphinato)zinc(II)] compounds having a variety of aromatic chromophores as a π-spacer, one of which was a pentacene, namely compound **64** (Scheme 6) [77]. The synthesis of **64** was accomplished by carrying out an *in situ* desilylation of **65** followed by Sonogashira-type coupling reaction with bromide **66** to afford **64**. Pentacene **64** had UV–vis absorptions in the Q-band region at 555 nm and 823 nm (λ_max_) in THF, with Soret bands at 421 and 493 nm. The band gap was determined from the UV–vis absorption edge to be 1.41 eV, which compared well with the electrochemically determined band gap of 1.40 eV measured in CH_2_Cl_2_.

**Table 1:** Properties of 6,13-disubstituted pentacenes with oligothiophenes.

Compound	λ_max_^[a]^ (in toluene) /nm	λ_max_ (thin film) /nm	*E*_g_^opt [b]^/eV	*E*_g_^electro [c]^/eV	*E*_HOMO_^[c]^/eV	*E*_LUMO_^[c]^/eV	*T*_d_^[d]^/°C	t_1/2_^[e]^/min
**27**	620	622	1.97	2.02	–5.26	–3.24	300	12
**59**	635	625	1.94	1.98	–5.24	–3.26	350	75
**60**	639	633	1.93	1.75	–5.16	–3.41	340	54
**61**	628	633	1.96	2.08	–5.22	–3.14	360	39
**62**	638	673	1.93	1.89	–5.16	–3.27	375	97
**63**	643	686	1.92	1.79	–5.14	–3.35	380	50

^[a]^ Lowest-energy absorption maximum. ^[b]^ Optical band gap (*E*_g_^opt^) determined from the onset of the lowest-energy visible absorption band. The onset was defined as the intersection between the baseline and the tangent line that touches the point of inflection. ^[c]^ *E*_g_ = *E*_LUMO_ – *E*_HOMO_ = (*E*_1/2_^red^ + 4.44) – (*E*_1/2_^ox^ + 4.44). ^[d]^ Decomposition temperatures determined by TGA analysis. ^[e]^ Half-life times (t_1/2_) for the photooxidative stability determined by monitoring the decrease in absorbance at λ_max_ for an air-saturated solution of the pentacene (1.0 × 10^–4^ M) in THF under ambient light at 22 °C and fitting the data to a unimolecular first order kinetics.

Very recently, Lin and coworkers have synthesized conjugated pentacene-porphyrin derivative **67** following a route analogous to Therien (Scheme 6), starting from porphyrin bromide **68** as the coupling partner [78]. UV–vis spectroscopic study of **67** (in THF) reveals Soret bands at 429 nm and 482 nm and an extremely broadened Q-band in the 500–800 nm region (λ_max_ = 751 nm). Intramolecular charge transfer is tentatively assigned for the lowest absorption band because it is significantly red-shifted in comparison to the absorption spectra of the components. Pentacene **67** emits at λ_max,em_ = 778 (in THF) using λ_exc_ = 429 nm. Electrochemical study of **67** reveals that oxidation is irreversible (no potential was given), while quasi-reversible reduction is observed at –0.85 V and –1.10 V *vs.* standard calomel electrode (SCE). The first reduction potential of **67** is closer to that of pentacene precursor **65** (0.92 V *vs.* SCE) than the reduction of model porphyrin **69** (–1.23 V *vs.* SCE) suggesting the reduction is occurring at the pentacene moiety. The photovoltaic properties of **67** have been studied, and, despite having a broad absorption spectrum able to capture a significant portion of the solar output spectrum, devices based on **67** have lower overall efficiencies (0.10%) than related materials in which the pentacene moiety is formally replaced with an anthracene moiety (which had an overall efficiency of 5.44%). The poor performance of sensitized solar cells based on pentacene **67** has been attributed to rapid non-radiative relaxation from the singlet excited state.

### 3.2. Unsymmetrically 6,13-Disubstituted Pentacenes

**Scheme 7:**
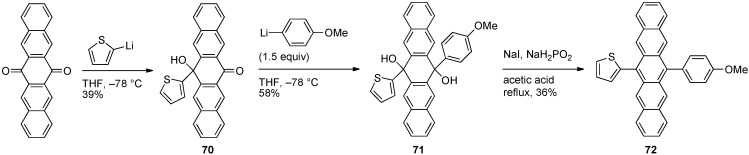
Synthesis of unsymmetrical pentacene **72**.

As can be seen from the synthesis of unsymmetrical pentacene **67** just described above (Scheme 6), unsymmetrically 6,13-disubstituted pentacenes can be challenging to synthesize and a general approach to such compounds would be synthetically beneficial. The synthesis of unsymmetrically 6,13-disubstituted pentacenes can be envisioned via the addition of two different nucleophiles in a stepwise fashion to 6,13-pentacenequinone. In the method reported by Dehaen and coworkers (Scheme 7) [44], addition of 2-lithiothiophene (as the limiting reagent) to a suspension of 6,13-pentacenequinone in THF at –78 °C afforded the monoaddition product **70** in 39% yield. Addition 1.5 equiv of the second nucleophile to mono-ketone **70** afforded unsymmetrical diol **71** in 58% yield. This diol was aromatized using NaI and NaH_2_PO_2_ in the presence of acid to afford unsymmetrical pentacene **72** in 8% overall yield.

In 2008, a general stepwise approach to unsymmetrical pentacene chromophores was reported by Tykwinski and Lehnherr, providing electronically varied pentacenes **73–81** in high yields over three steps (Table 2) [79]. Key to this stepwise protocol was the slow addition of one equivalent of LiC≡CSi*i*‑Pr_3_ at –78 °C to a suspension of 6,13-pentacenequinone at room temperature. This allowed for the continued dissolution of 6,13-pentacenequinone (as the dissolved portion is being consumed) to compete effectively against the addition of a second acetylide into the highly soluble lithiated alkoxide intermediate. With **82** in hand, the subsequent addition of two or more equivalents of the second acetylide (as the first equivalent is consumed by the acidic alcohol proton of **82**) resulted unsymmetrical diol **83**. The addition of the two different nucleophiles to 6,13-pentacenequinone can be combined into a one-pot procedure, providing **83** (with R = Ph) in comparable yields (84% yield for the 1-step synthesis of **83** *vs.* 77% for the 2-step route) [80]. Unsymmetrical diols **83** were aromatized at room temperature using SnCl_2_·2H_2_O to afford polarized pentacenes **73–81** in which either an electron-donating group or electron-withdrawing group could be incorporated to vary the electronic properties of pentacene (see Table 2). It should be noted that the Sn^II^-mediated aromatization to **73–81** can be carried out without using an additional Brønsted acid (e.g., HCl, H_2_SO_4_, AcOH), and the milder conditions provide excellent yields for a wider substrate scope [29,30,79,80].

**Table 2:** Pentacenes dyads and reported optical properties. 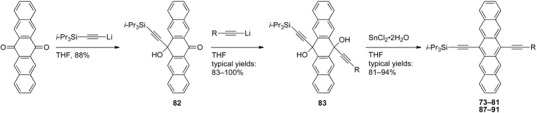

Compound number and R =		λ_mid_^[a]^ (CH_2_Cl_2_)/nm	λ_max_^[b]^ (CH_2_Cl_2_)/nm	λ_max_ (film)/nm	λ_max,em_ (CH_2_Cl_2_)/nm	Φ_F_^[c]^ (CH_2_Cl_2_)	*E*_g_^opt [d]^ (CH_2_Cl_2_)/eV	*E*_g_^electro [e]^ (3:1 PhH/MeCN) /eV	*T*_d_ (DSC)/°C
**1**	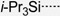	328	643	–	649	0.15	1.84	–	265
**73**	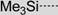	327	642	–	649	0.13	1.84	–	167
**74**	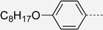	368	656	–	666	0.05	1.78	–	136
**75**	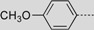	367	655	–	666	0.05	1.78	–	149
**76**	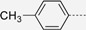	363	654	–	661	0.10	1.80	–	191
**77**	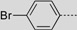	366	654	–	663	0.12	1.80	–	214
**78**	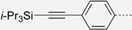	364	659	–	668	0.12	1.78	–	210
**79**	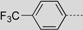	365	654	–	667	0.06	1.79	–	215
**80**	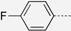	357	651	–	660	0.13	1.81	–	194
**81**	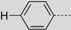	360	652	671	661	0.13	1.81	1.83	180
**86**	(see Scheme 8)	374	664	–	679	0.04	1.76	–	175
**87**	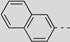	381	657	679	667	0.12	1.79	1.80	197
**88**	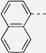	395	658	679	668	0.12	1.79	1.81	210
**89**	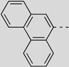	398	660	686	670	0.11	1.78	1.79	221
**90**	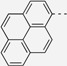	440	671	712	681	0.07	1.74	1.74	256
**91**	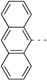	465	671	712	688	0.006	1.74	1.71	271

^[a]^ Wavelength of most intense absorption in the range of 325–500 nm. ^[b]^ Lowest-energy absorption maxima. ^[c]^ Measured using λ_exc_ = 551 nm and cresyl violet perchlorate as a standard (see reference [83]). ^[d]^ The wavelength used as the absorption edge for determining *E*_g_^opt^ corresponds to the lowest-energy absorption that has a molar absorptivity *ε* ≥ 1000 L·mol^–1^·cm^–1^. ^[e]^ *E*_g_^electro^ determined from the separation between the first oxidation and first reduction potentials measured in benzene/MeCN (3:1 v/v) containing 0.1 M *n*-Bu_4_NPF_6_ as supporting electrolyte.

**Scheme 8:**
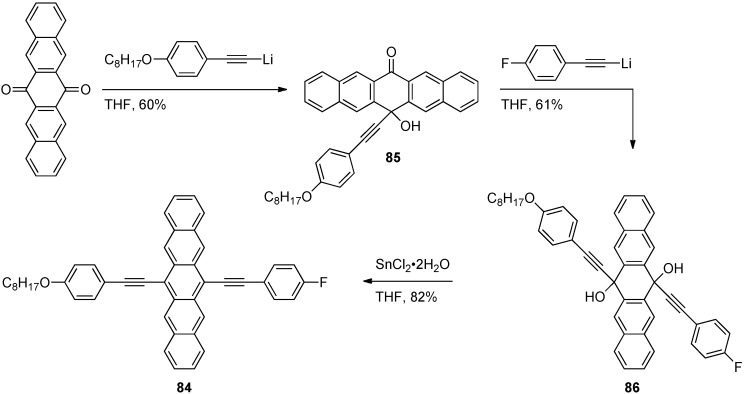
Synthesis of push-pull pentacene **84**.

Using a stepwise approach, Tykwinski and Lehnherr synthesized polarized pentacene **84** (Scheme 8) [79]. Addition of one equivalent of *p*-octyloxybenzene Li-acetylide into 6,13-pentacenequinone afforded monoaddition product **85** in 60% yield. Addition of excess *p*-fluorobenzene Li-acetylide to a solution of **85** afforded unsymmetrical diol **86** in 61% yield, which was aromatized using SnCl_2_·2H_2_O to provide pentacene **84** in 82% yield. Push-pull pentacene **84** has an absorption λ_max_ of 664 nm (in CH_2_Cl_2_), which is more red-shifted than any of the unsymmetrical derivatives **73–81**.

Ideally, organic materials for solar cell applications would absorb light throughout the solar spectrum [35,81,82]. This requires materials with electronic absorption spectra covering a wide range of energies across the UV–vis spectrum and into the near-IR. Unfortunately, functionalized pentacenes such as 6,13-bis(triisopropylsilylethynyl)pentacene (**1**) typically have a very narrow absorption region in the ultra-violet region (centered around ca. 310 nm) in addition to absorption bands in the visible region of 525–660 nm; they are quite transparent in the region of 350–525 nm [20]. Thus, a homologous series of pentacene–PAH dyads (**87–91**, Table 2) has been realized toward maximizing absorption [84].

**Figure 8:**
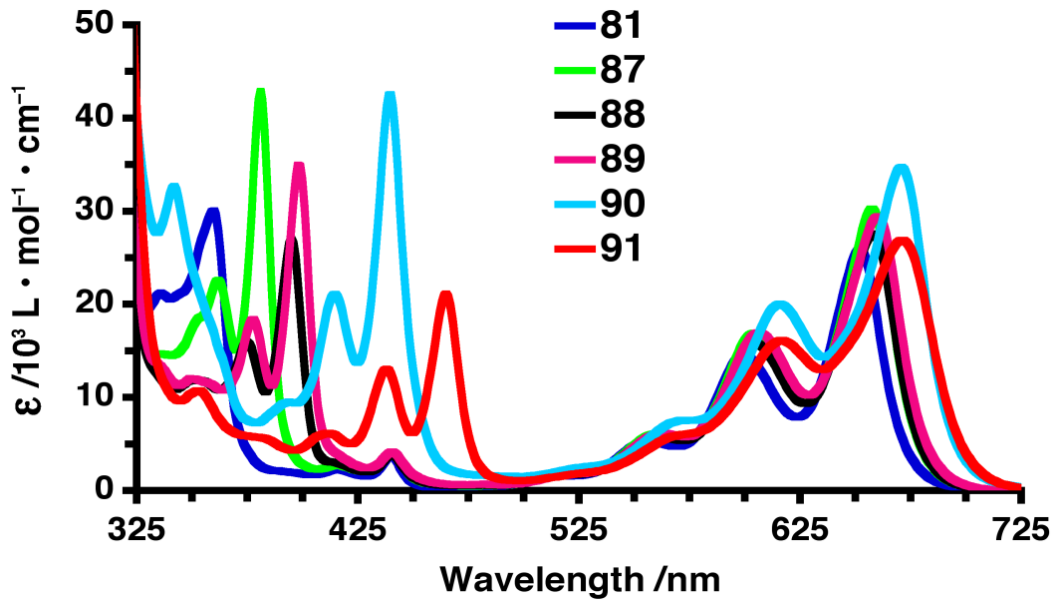
UV–vis absorption spectra of pentacenes **81** and **87–91** in CH_2_Cl_2_.

The optical properties of these pentacene-PAH dyads have been characterized by UV–vis absorption and emission spectroscopy. UV–vis spectroscopy (in CH_2_Cl_2_) shows that the ethynyl linker in **87–91** facilitates electronic communication between the PAH pairs, as observed by the red shift in the observed λ_max_ values for **87–91** as the pendent PAH chromophore is formally increased in size (Table 2). More interesting effects are observed in the mid-energy absorption range between 325–500 nm, as absorptions (λ_mid_) are substantially red-shifted with increasing size of the pendent PAH group (Figure 8). Thus, the structural evolution in the acene series from phenyl **81** (λ_mid_ = 360 nm) to naphthyl **88** (λ_mid_ = 395 nm) to anthryl **91** (λ_mid_ = 465 nm) demonstrates a red shift of over 100 nm for this absorption. Fluorescence quantum yields range from 0.11–0.13 for dyads **81**, and **87–89**, and then drop to 0.07 for pyrenyl **90**. Anthryl dyad **91** has a very low Φ_F_ = 0.006, approaching the behavior of non-emissive conjugated pentacene dimers (*vide infra*). Cyclic voltammetry (CV) experiments suggest that the energy of the HOMO is raised while the LUMO energy is lowered as benzannulation is increased from **87** to **91**. Furthermore, *E*_g_ values estimated from both UV–vis and CV analysis consistently decrease as annulation increases.

**Figure 9:**
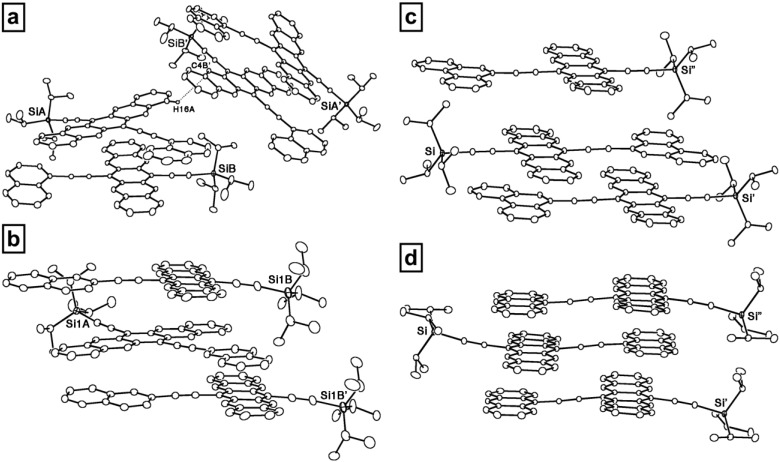
ORTEP representation illustrating solid-state packing of (a) **88**, (b) **87**, (c) **89**, and (d) **91**. Figure adapted with permission from reference [84]. Copyright 2009 Wiley-VCH Verlag GmbH & Co. KGaA.

Solid-state analysis of pentacene-based PAH dyads **87–89** and **91** reveals that the molecules are able to π-stack with a cofacial arrangement with short interplanar distances (3.3–3.5 Å). The choice of the PAH moiety, as well as the point of attachment of 6,13-diethynylpentacene, offers the ability to manipulate the solid-state packing. X-ray crystallography nicely shows that with increasing size of the pendent PAH group in **87–89** and **91**, cofacial packing is maximized between nearest neighbors (Figure 9). This fact should ultimately provide for a larger transfer integral, and, thus, enhanced conduction and improved charge-carrier mobility [85].

In addition to the pentacene-based PAH conjugates discussed above, there are of course the pentacenes which have aromatic rings fused to their skeleton, for example, hexacenes [22,86], heptacenes [22,23,86,87,88], and a recently reported functionalized nonacene [89]. Additional examples exist with pentacene structures that have one or more thiophene ring(s) fused to the pentacene core, such as pentadithiophenes (which contain 7-linearly fused aromatic rings) [90] and pentacenothiophene (which contains 6-linearly fused aromatic rings) [91]. A hexathiapentacene has also been formed and displays interesting properties [92,93,94].

## 4. Conjugated Pentacene Oligomers

**Scheme 9:**
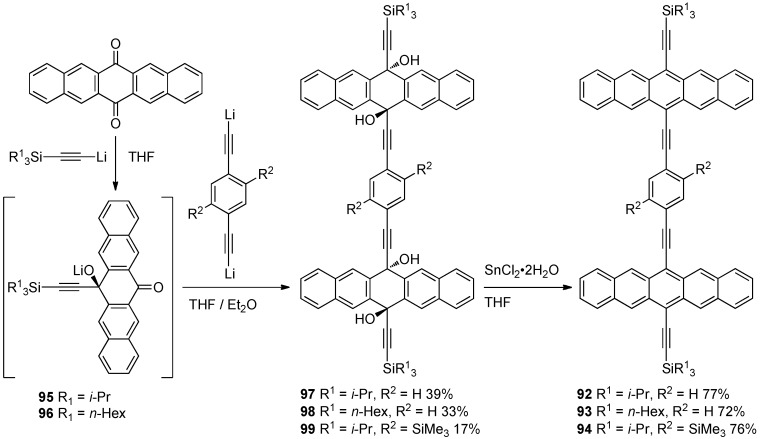
Synthesis of conjugated pentacene dimers **92–94**.

Conjugated pentacene dimers **92–94** were first reported in 2008 [80]. The synthesis was accomplished by addition of either *i*-Pr_3_Si-acetylide or *n*-Hex_3_Si-acetylide to 6,13-pentacenequinone to achieve intermediates **95** and **96**, respectively, followed by reaction with a dilithiated 1,4-diethynyl-benzene to provide tetraols **97–99** (Scheme 9). Sn^II^-mediated reductive aromatization of tetraols **97–99** afforded pentacene dimers **92–94**. Increased solubility was achieved by formally replacing the *i*-Pr_3_Si endgroups of **92** with *n*-Hex_3_Si groups in **93**. Conjugated pentacene dimers **92–94** had a UV–vis absorption λ_max_ around 680 nm (in CH_2_Cl_2_), a red shift of ca. 30 nm compared to **81**. These conjugated dimers were found to have insignificant emission in CH_2_Cl_2_.

**Figure 10:**
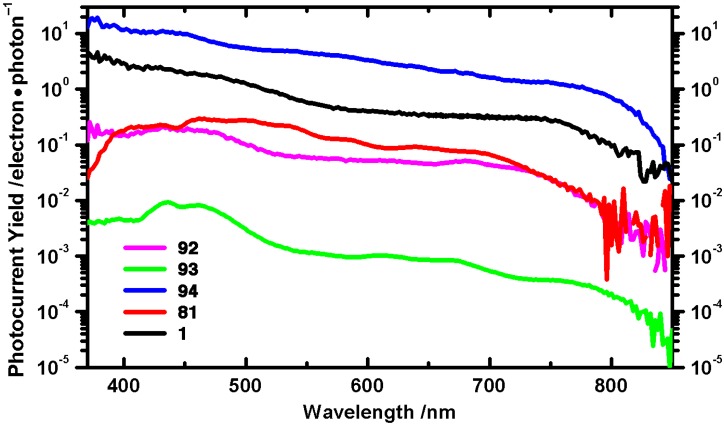
Photocurrent yield (370–850 nm) for dimers **92**–**94** and related monomers **1** and **81**. Figure adapted with permission from reference [80]. Copyright 2008 American Chemical Society.

Thin film photodetectors have been fabricated from dimers **92–94**, and photoconduction has been measured in comparison to 6,13-bis(triisopropylsilylethynyl)pentacene (**1**). The photocurrent yield for dimer **92** (Figure 10) is in the same order of magnitude as pseudo-monomer **81**, while dimer **93** has the lowest efficiency, presumably due to the larger size of the pendent *n*-Hex_3_Si groups that can disrupt intermolecular interactions between pentacene chromophores. Dimer **94** has the highest efficiency, with bulk photoconductive gain >10, *i.e.*, for every photon absorbed more than 10 charge-carriers traverse the active region of the device [95]. Thus, **94** outperforms **1**, which has become somewhat of a benchmark in the field [17,18,20]. Furthermore, this efficiency places spin-cast films of dimer **94** within the same order of magnitude as thermally deposited films of pristine pentacene which has a photoconductive gain >16 [96].

**Scheme 10:**
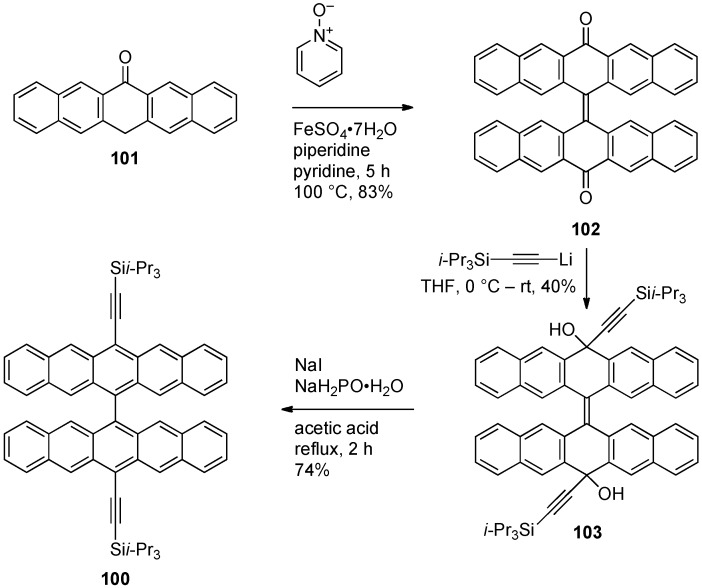
Synthesis of 13,13'-bis(triisopropylsilylacetylene)-6,6'-dipentacenyl **100**.

In 2009, Wu and coworkers reported the synthesis of pentacene dimer **100** (Scheme 10) [97]. The synthesis was accomplished by dimerization of 6-pentacenone (**101**) using pyridine *N*-oxide in the presence of FeSO_4_ to form 6,6'-bispentacenequinone **102** in 83% yield. Addition of a Li-acetylide to diketone **102** afforded diol **103** which was aromatized using NaI and sodium hypophosphite to pentacene dimer **100** in 74% yield. Dimer **100**, a deep blue solid, has UV–vis absorption λ_max_ of 637 nm in CHCl_3_, slightly blue-shifted from pentacene **1** (λ_max_ = 642 nm in CHCl_3_). Thin films of **100** have a minimally red-shifted λ_max_ of 11 nm compared to solution-state data. The half-life of toluene solutions of pentacene **100** under white light irradiation (100 W) has been determined to be 350 min, compared to 140 min for **1** when measured under the same conditions. X-ray crystallographic data reveals that the two pentacene moieties of dimer **100** are nearly orthogonal to each other (78° twist), and that each pentacene moiety is part of separate cofacial π-stacking interactions with interplanar distances of ca. 3.4 Å. This results in two π-stacking axes nearly orthogonal to each other. FET were fabricated by using vapor-deposition to form thin films of **97** onto octadecyltrichlorosilane-treated Si/SiO_2_ substrate heated at 200 °C, yielding mobilities as high as 0.11 cm^2^·V^–1^·s^–1^ when measured in the saturated regime [98].

**Scheme 11:**
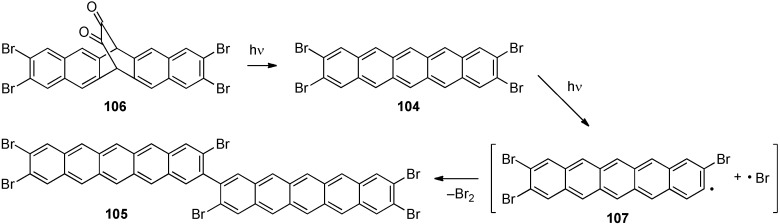
Photochemical synthesis of conjugated pentacene dimer **105**.

In 2009, Neckers reported the photochemical dimerization of 2,3,9,10-tetrabromopentacene (**104**) to produce 3,3',9,9',10,10'-hexabromo-2,2'-bipentacene (**105**, Scheme 11) [99,100]. Photochemically induced decarbonylation of **106** produced **104**, and upon prolonged irradiation the dimerization of **104** produced **105** (confirmed by MALDI-TOF MS). The mechanism was postulated to proceed via formation of pentacene radical **107** (via loss of Br•) followed by bimolecular recombination of **107**. Little is known about dimer **105** other than its constitution as established by MALDI-TOF MS and a tentative λ_max_ value of ca. 688 nm (in toluene).

**Figure 11:**
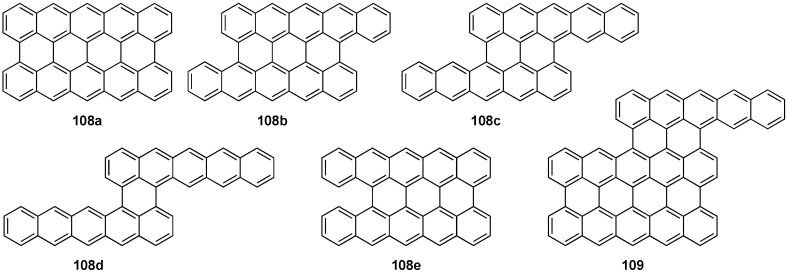
Proposed structures for **108a–e** and **109**.

The purification of pentacene via sublimation is known to cause the decomposition of pentacene into a number of interesting byproducts depending on the nature of the atmosphere used for the purification [101]. The reactivity of the central ring with oxygen, water, or hydrogen is documented by the formation of 6,13-pentacenequinone and 6,13-dihydropentacene depending on the nature of the carrier gas and its purity when performing physical vapor transport for the growth of pentacene crystals [101,102]. The residue material from the sublimation contains compounds with the proposed structure **108a** (peripentacene) and **109** (trisperipentacene) as determined by laser desorption FTMS (Figure 11) [102]. The MS data features a distribution of peaks around the expected signal of *m*/*z* 546 for **108a**, which is explained by the relative positioning of the two pentacene molecules to one another. In the case that the two pentacenes are offset, one or more unfused position(s) remain, and this would result in a molecular mass increase of *m*/*z* 2 (**108b**), 4 (**108c**), or 6 (**108d**). The offset between the two pentacene moieties is not necessary, and these signals may be due to unfused sites such as in structure **108e**; a number of isomers can be imagined. No experimental evidence has conclusively determined the exact structures related to these signals, but a theoretical computational study has been carried out [103]. Likewise, the signal at *m*/*z* 818 could be due to one of a number of possible isomers, such as **109**, which has two unfused positions (*i.e.*, [C_66_H_26_]^+^). These materials have not been thoroughly characterized, but the authors claim they are "…more conductive than pentacene, with a linear I–V dependence similar to graphite" [102].

## 5. Conjugated Pentacene Polymers

**Scheme 12:**
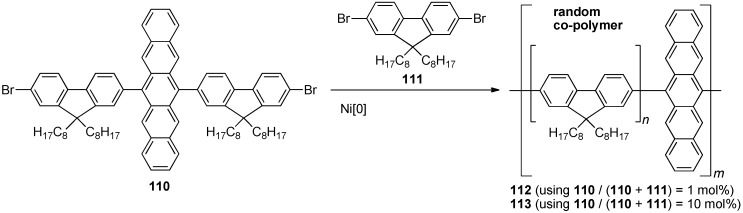
Synthesis of conjugated random copolymers **112–113** containing pentacene.

In 2001, Tokito and coworkers have reported the first pentacene polymers [104]. Ni[0]-mediated Yamamoto coupling of dibromo monomers **110** and **111** provides random copolymers **112** and **113** depending on the relative mole ratio of the monomers used (Scheme 12). Polymer **112** has a weight-average molecular weight (*M*_w_) of 86,000 g·mol^–1^ and polydispersity (PDI) of 1.7, while increasing the pentacene content results in polymer **113** with a lower MW of 61,000 g·mol^–1^ and smaller PDI of 1.6. Thin films of polymer **113** have an absorption λ_max_ at 623 nm and emission at λ_max,em_ = 625 nm (excitation at 365 nm). Polymer light emitting diodes (PLEDs) have been constructed using the following arrangement: ITO/PEDOT/polymer/Ca/Al. PLEDs with polymer **113** show a turn-on voltage of 30 V, a maximum photoluminescence of 240 cd/m^2^, and photometric efficiencies of 0.11 cd/A when operated at 100 cd/m^2^. The turn-on and operating voltages for **113** are higher than those of the homopolymer (polyfluorene). The incorporation of the pentacene into the polymer is believed to create electron- or hole-carrier traps, which likely cause a decrease in the carrier mobility. By decreasing the content of pentacene in the polymer, *i.e.* going from **113** to **112**, turn-on voltages are reduced to 11 V and maximum luminance and photometric efficiencies improved to 1020 cd/m^2^ and 0.32 cd/A, respectively. It is noted that the PLEDs had a half-life of less than 10 h, which is insufficient for practical applications.

**Scheme 13:**
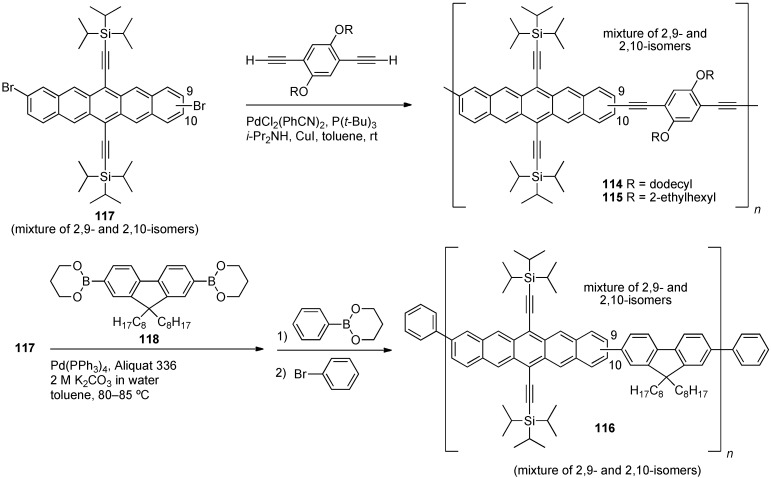
Synthesis of conjugated pentacene-polymers **114–116**.

In 2007, Okamoto and Bao reported conjugated polymers **114–115** [105], which was followed by a 2008 report of a related polymer (**116**, Scheme 13) [106]. Dibromopentacene monomer **117** was used as a regio-mixture of 2,9- and 2,10-dibromopentacene to form copolymers **114–116** via a Sonogashira-type coupling with 1,4-diethynylbenzenes. Similarly, polymer **116** was formed from dibromides **117** via Suzuki coupling with fluorene diboronic ester **118**, followed by endcapping of the polymer with phenyl groups.

Polymer **114** has a number-average molecular weight (*M*_n_) = 1.13 × 10^4^ g·mol^–1^ and PDI = 2.21, while polymer **115** has *M*_n_ = 2.39 × 10^4^ g·mol^–1^ and PDI = 2.42, while polymer **116** has *M*_n_ = 3.64 × 10^4^ g·mol^–1^ and PDI = 3.21. Polymer **115** shows greater solubility (>5.0 mg/mL in chlorinated solvents) compared to **114** (~0.3 mg/mL) which is likely due to the increased branching of the alkoxy group on the aryl spacer. Polymer **116** is soluble in halogenated benzenes, such as *o*-dichlorobenzene (*o*-DCB) with solubility of ~5.0 mg/mL. DSC analysis of polymers **114–115** shows no thermal transitions between 25 and 350 °C, while TGA data for polymers **114–116** reveals thermally induced weight loss occurs around 400 °C. Polymer **115** has better solution-state photochemical stability in comparison to 6,13-bis(triisopropylsilylethynyl)pentacene (**1**) as observed by monitoring the decrease in absorption at λ_max_ in the UV–vis spectra over time. This is attributed to the slightly lower energy HOMO (by 0.04 eV) in polymer **115** compared **1**, making the polymer harder to oxidize (Table 3). Fabrication of bulk heterojunction solar cells with polymer **116** and PCBM has been attempted, but result in "poor performance" [106]. This may be due to the high reactivity of pentacenes with C_60_ and its derivatives which would destroy the pentacene chromophore [107,108,109,110,111].

**Table 3:** Optical and electrochemical properties of polymer **114–116** and compared to monomer **1**.

Compound	*E*_red1_^onset[c]^/V	*E*_red2_^onset[c]^/V	*E*_ox_^onset[c]^/V	λ_max_, λ_onset_ (*o*-DCB) /nm	λ_max_, λ_onset_ (film) /nm	λ_max,em_ (*o*-DCB) /nm	*E*_g_^electro^/eV	*E*_g_^opt[c]^/eV
**114**	–1.37	–1.80	0.32	671, 693	672, 736	676^[a]^	1.69	1.68
**115**	–1.42	–1.82	0.28	674, 695	674, 706	679^[a]^	1.70	1.76
**116**	–1.40	–1.89	0.24	675, 695	670, 695	683^[b]^	1.64	1.78

^[a]^ λ_exc_ = 435 nm. ^[b]^ λ_exc_ = 412 nm. ^[c]^ Potentials measured *vs.* ferrocenium/ferrocene (Fc^+^/Fc) couple in *o*-DCB containing 0.05 M *n*-Bu_4_NPF_6_ as supporting electrolyte.

## 6. Conclusions

An array of functionalized pentacenes has been synthesized over the past decade, some of which have provided useful devices. Pentacene oligomers and polymers, on the other hand, are still a relatively unexplored research area with much promise for interesting properties. Much the same as functionalization of monomeric pentacene-based materials has afforded the ability to vary electronic properties and control solid-state organization, oligomers and polymers offer many of the same opportunities. The incorporation of pentacene and related chromophores into an oligo- or polymer framework is, however, a very young field and many discoveries await the hands and imagination of talented synthetic chemists.
